# Di̇agnosti̇c yi̇eld of EBUS i̇n medi̇asti̇nal stagi̇ng of extrapulmonary mali̇gnanci̇es; si̇ngle center cli̇ni̇cal experi̇ence

**DOI:** 10.1186/s12890-026-04273-4

**Published:** 2026-04-15

**Authors:** Mehmet Akif Ekici, Oguzhan Turan, Enes Fatih Çelik, Mehmet Akif Tezcan, İbrahim Ethem Özsoy, Bayram Metin

**Affiliations:** Department of Thoracic Surgery, Kayseri City Hospital, Kayseri, Türkiye

**Keywords:** EBUS, Extrapulmonary Malignancies, Mediastinal Staging

## Abstract

**Background:**

Accurate mediastinal staging is essential for treatment planning and prognostic assessment in patients with extrapulmonary malignancies. Endobronchial ultrasonography (EBUS) has emerged as a minimally invasive modality for mediastinal evaluation; however, evidence regarding its diagnostic performance in extrapulmonary malignancies remains limited.

**Methods:**

Patients with known extrapulmonary malignancies who underwent EBUS for mediastinal staging were retrospectively analyzed. Demographic characteristics, radiological findings, sampled lymph node stations, and pathological results were evaluated. Factors potentially associated with malignant mediastinal involvement were assessed.

**Results:**

EBUS enabled successful sampling of mediastinal and hilar lymph nodes across multiple stations. Malignant involvement was identified in a subset of patients, while benign etiologies—including reactive and granulomatous lymphadenopathy—accounted for the majority of cases. Increased lymph node diameter was associated with malignancy, whereas primary tumor type and the number of sampled lymph nodes were not independent predictors of malignant pathology.

**Conclusions:**

EBUS appears to be a safe and reliable tool for mediastinal staging in patients with extrapulmonary malignancies. Its minimally invasive nature and diagnostic performance support its use as a first-line modality, potentially reducing the need for confirmatory mediastinoscopy in selected patients.

## Background

Endobronchial ultrasonography (EBUS) is widely used in the diagnosis of mediastinal diseases and in the staging of both pulmonary and extrapulmonary malignancies. Although mediastinoscopy remains the gold standard for the diagnosis of mediastinal diseases, EBUS has become routinely implemented in clinical practice due to its low mortality and morbidity rates, minimally invasive nature, short procedure time, and the advantage of early discharge, while maintaining a high diagnostic yield [1]. 

Similar to mediastinoscopy, EBUS allows sampling of the upper paratracheal (stations 2R/2L), lower paratracheal (stations 4R/4L), and subcarinal (station 7) mediastinal lymph node regions. Unlike mediastinoscopy, EBUS also enables sampling of peribronchial lymph node stations, including stations 10, 11, and 12. In cases of negative EBUS findings, the European Society of Thoracic Surgeons guidelines still recommend confirmatory video mediastinoscopy [2, 3]. However, the necessity of confirmatory mediastinoscopy remains controversial, and several studies suggest that it may be omitted following negative EBUS results [4, 5]. 

In patients with extrapulmonary malignancies, mediastinal lymph nodes with pathological size and increased metabolic activity are frequently detected on positron emission tomography (PET) scans performed for initial staging or post-treatment surveillance. However, these lymph nodes are often enlarged due to inflammatory or infectious etiologies rather than metastatic involvement [6]. 

Accurate mediastinal staging in this patient population is crucial for prognostic assessment and treatment planning. Nevertheless, data evaluating the role of EBUS in mediastinal staging of extrapulmonary malignancies remain limited [7, 8]. 

The aim of this study was to evaluate the diagnostic performance based on follow-up of EBUS for mediastinal staging in patients with extrapulmonary malignancies.

## Methods

This is a retrospective study. According to our institutional protocol, patients scheduled for EBUS underwent preoperative complete blood count, coagulation tests, routine biochemical analyses, and anesthesiology consultation. The procedure was performed after an 8-hour fasting period. All procedures were carried out under general anesthesia using a laryngeal mask airway, with ventilation maintained through the LMA channel.

The EBUS device used was the Fujifilm EB-530US. Sampling was performed using an Olympus ViziShot EBUS-TBNA needle, and an Olympus balloon was used during the procedure. Lymph node selection for EBUS-TBNA was based on preprocedural thoracic computed tomography and/or PET-CT findings. Nodes with a short-axis diameter of ≥ 10 mm, increased FDG uptake, or suspicious ultrasonographic features were preferentially sampled. When multiple stations were accessible, sampling was prioritized according to the radiologic suspicion and technical feasibility of the procedure. Each target lymph node station was sampled with at least 3 needle passes, and additional passes were performed when the obtained material was considered macroscopically insufficient. Rapid on-site evaluation (ROSE) was not available at our institution. Aspirated material was processed for cytology plus cell-block preparation. Metastatic involvement was diagnosed on the basis of cytomorphologic findings and, when needed, immunohistochemical markers compatible with the known primary malignancy. Postoperative follow-up was conducted either in the ward or in the intensive care unit according to the patient’s clinical condition, and all patients were hospitalized following the procedure [9]. 

A total of 370 patients who underwent EBUS at our center between November 2023 and December 2025 were retrospectively screened. Among these, 69 patients with a known extrapulmonary malignancy who underwent EBUS for mediastinal staging were included in the study. Four patients were excluded due to insufficient biopsy material on pathological examination.

Pathological findings and clinical characteristics were obtained from the hospital database. Patients were divided into two groups based on EBUS pathology results: benign and malignant. The relationship between the localization of the primary malignancy and the sampled lymph node stations was analyzed within each group.

For patients with negative EBUS findings, follow-up data were reviewed to assess the reliability of benign cytopathologic results. Follow-up consisted of clinical evaluation and radiologic imaging with thoracic CT and/or PET-CT performed at 3–6 month intervals according to oncologic surveillance protocols. The median follow-up duration was 6 months (range, 1–16 months). Negative EBUS findings were considered clinically reliable in the absence of radiologic progression, newly developed mediastinal FDG uptake, or pathologically confirmed mediastinal metastasis during follow-up.

Statistical analyses were performed using SPSS version 27 (IBM Corp.). Continuous variables were compared using the independent samples t-test, and categorical variables were analyzed using the chi-square test. Binary logistic regression analysis was performed to evaluate factors associated with malignant mediastinal involvement. The multivariable model included radiological lymph node diameter, breast cancer status, and head and neck cancer status. Odds ratios (ORs), 95% confidence intervals (CIs), and p-values were reported. Given the limited number of malignant events, the model was kept parsimonious to reduce the risk of overfitting. A *p*-value < 0.05 was considered statistically significant.

## Results

Of the 69 patients evaluated, four were excluded due to insufficient sampling, and the final analysis included 65 patients. Among these, 18 patients (27%) had malignant pathology and 47 patients (73%) had benign pathology. The mean age of the cohort was 62.9 ± 9.3 years, with no statistically significant difference between the malignant and benign groups. Gender distribution was also similar between groups. Table 1 summarizes the characteristics of the final study cohort after exclusion of four patients with inadequate sampling. All percentages in the revised tables were recalculated according to the final analytic sample or the relevant subgroup denominator, as applicable. (Table 1)

**Table 1:** Clinical and demographic characteristics of patients

	*n*	%	Mean ± SD (Min–Max)
Primary Malignancy			
Gastrointestinal System	19	29.2	
Breast	20	30.8	
Head and neck	8	12.3	
Lymphoproliferative Disease	6	9.2	
Others	12	18.5	
Age			63.11 ± 9 (38–84)
Gender			
Male	30	46.2	
Female	35	53.8	
Radiological Lymph Node Diameter			20.65 ± 10 (6–50)
Pathology Result			
Metastasis	18	27.7	
Granulomatous Disease	14	21.5	
Reactive/Anthracotic	33	50.8	
Biopsy Stations			
2R	2	3.1	
4R	34	49.2	
4L	2	3.1	
7	40	61.5	
10R	2	3.1	
10L	2	3.1	
11R	15	23.1	
11L	6	9.2	

The mean radiological lymph node diameter was significantly greater in the malignant group compared to the benign group (24.0 ± 8.8 mm vs. 19.3 ± 11.3 mm, *p* = 0.042). ROC analysis showed that radiological lymph node diameter had a modest but statistically significant discriminative ability for predicting malignant mediastinal involvement (AUC = 0.664, 95% CI 0.529–0.799, *p* = 0.042). The optimal cut-off value based on the Youden index was 18 mm, yielding a sensitivity of 77.8% and a specificity of 53.2%.

No statistically significant differences were observed between groups regarding primary malignancy subtypes. Malignancy was detected in 40% of patients with breast cancer and in 50% of patients with head and neck cancer; however, these differences were not statistically significant. Multivariable logistic regression analysis was performed including head and neck cancer, breast cancer, and radiological lymph node diameter. The overall model was statistically significant (omnibus test: χ² = 8.088, df = 3, *p* = 0.044) and showed acceptable calibration (Hosmer–Lemeshow test: *p* = 0.707). However, none of the individual variables reached independent statistical significance in the multivariable model. Radiological lymph node diameter was not an independent predictor of malignant mediastinal involvement (OR 1.040, 95% CI 0.987–1.096, *p* = 0.144). Likewise, breast cancer and head and neck cancer were not independently associated with malignant EBUS pathology (*p* = 0.057 and *p* = 0.052, respectively), although both showed borderline trends.

The most frequently sampled lymph node stations were station 7 (60.9%), station 4R (49.3%), and station 11R (24.6%). There were no statistically significant differences in malignancy rates among lymph node stations. Although a higher malignancy rate was observed at station 10 L, statistical significance was not consistently demonstrated, likely due to small sample sizes.

The total number of sampled lymph nodes did not differ significantly between the malignant and benign groups, indicating that the number of samples did not influence malignancy detection rates. Table 2 shows the characteristics of the benign and malignant groups. (Table 2)

**Table 2:** Clinical Distribution Of Groups

	Malign (*n* = 18)	Benign (*n* = 47)	*p*
Age (years, Mean ± SD)	59.7 ± 9.35	64.4 ± 9.07	0.069^a^
Gender			0.467ᶜ
Male	7 (38.9%)	23 (48.9%)	
Female	11 (61.1%)	24 (51.1%)	
Radiological LAP Diameter (mm ± SD)	24.0 ± 8.84	19.3 ± 11.32	0.042*ᵇ
Primary Malignancy Subtypes			0.21ᶜ
· Breast cancer	8 (44.4%)	12 (25.5%)	0.14ᵈ
· Head and neck cancer	4 (22.2%)	4 (8.5%)	0.14ᵈ
· Gastrointestinal malignancy	3 (16.7%)	16 (34.0%)	—
· Others	2 (11.1%)	10 (21.3%)	—
· Lymphoproliferative disease	1 (5.6%)	5 (10.6%)	—
EBUS Biopsy Stations			> 0.05ᶜ
· 2R	2 (11.1%)	0 (0.0%)	
· 4R	9 (50.0%)	23 (48.9%)	
· 4L	1 (5.6%)	1 (2.1%)	
· 7	10 (55.6%)	30 (63.8%)	
· 10R	1 (5.6%)	1 (2.1%)	
· 10L	2 (11.1%)	0 (0.0%)	
· 11R	2 (11.1%)	13 (27.7%)	
· 11L	2 (11.1%)	4 (8.5%)	
Total Number Of Lymph Nodes Sampled, Mean ± SD	1.61 ± 0.70	1.53 ± 0.58	0.645

^a^ Independent samples t-test

ᵇ ROC-related continuous variable comparison / independent samples t-test notation as used in the original table

ᶜ Chi-square test

ᵈ Fisher’s exact test

*p <0.05, which is statistically significant for Radiological LAP Diameter

## Discussion

In this retrospective study, 65 patients with extrapulmonary malignancies who underwent mediastinal staging with EBUS were evaluated. Patient age and gender distribution were comparable between malignant and benign groups, consistent with previous reports [10]. 

Two patients with negative EBUS findings but persistent radiological suspicion subsequently underwent mediastinoscopy, and both were found to be negative for malignancy. During follow-up, no mediastinal metastasis was detected in any patient. Among patients with persistent radiologic suspicion despite negative EBUS, two underwent confirmatory mediastinoscopy, and both had benign mediastinal pathology. These findings support the safety and reliability of EBUS for mediastinal staging in patients with extrapulmonary malignancies [4, 11]. 

The spectrum of extrapulmonary malignancies associated with mediastinal metastasis varies across studies, with breast cancer, gastrointestinal malignancies, head and neck cancers, malignant melanoma, and lymphomas most frequently reported [8, 12]. In the present study, breast cancer was the most common primary malignancy associated with mediastinal metastasis, which may be related to the preferential use of mediastinoscopy in certain malignancies such as lymphoma.

EBUS enables reliable sampling of upper and lower paratracheal, subcarinal, hilar, and peribronchial lymph nodes [13, 14]. In this study, the subcarinal station was the most frequently sampled site. Although no statistically significant differences were observed between stations, a relatively higher malignancy rate was noted at station 10L.

Lymph node size on computed tomography is commonly used to assess the likelihood of mediastinal malignancy [15]. Consistent with previous studies, increased lymph node diameter was significantly associated with malignancy in our cohort. However, the detection of metastasis in a 10-mm lymph node highlights the limitations of size-based criteria alone.

Mediastinal lymphadenopathy may result from various benign conditions [16]. In this study, granulomatous disease and reactive lymphadenopathy accounted for a substantial proportion of benign diagnoses. This finding may reflect the higher prevalence of granulomatous diseases in our region. In addition, granulomatous reactions related to chemotherapy and increased susceptibility to infections in patients receiving chemotherapy or radiotherapy may further contribute to lymph node enlargement [17, 18]. 

This study has several limitations. Its single-center design limits the generalizability of the findings, and multicenter studies are needed for validation. Patient selection by multiple clinicians may have introduced heterogeneity. The widespread use of PET-CT may have led to sampling of radiologically non-pathological lymph nodes, potentially increasing the proportion of benign findings. The absence of routine surgical confirmation represents an inherent limitation of retrospective studies; however, the lack of radiological or clinical evidence of mediastinal progression during follow-up provides reassurance regarding the reliability of negative EBUS findings.

## Conclusions

Endobronchial ultrasonography is a safe and reliable modality for mediastinal staging in patients with extrapulmonary malignancies. Its minimally invasive nature and ability to provide tissue diagnosis across multiple lymph node stations support its use as a first-line diagnostic approach. In selected patients, EBUS may reduce the need for confirmatory mediastinoscopy while facilitating accurate staging and treatment planning. 

## Data Availability

Data are available from the corresponding author on reasonable request.

## Abbreviations

EBUS: Endobronchial ultrasonography
PET: Positron emission tomography
LMA: Laryngeal mask airway
TBNA: Transbronchial needle aspiration
